# Develop to Term Rat Oocytes Injected with Heat-Dried Sperm Heads

**DOI:** 10.1371/journal.pone.0078260

**Published:** 2013-11-04

**Authors:** Kyung-Bon Lee, Ki-Eun Park, In-Kiu Kwon, Swamy K. Tripurani, Keun Jung Kim, Ji Hye Lee, Koji Niwa, Min Kyu Kim

**Affiliations:** 1 The Graduate School of Natural Science and Technology, Okayama University, Okayama, Japan; 2 Division of Animal and Nutritional Sciences, Laboratory of Animal Biotechnology and Genomics, West Virginia University, Morgantown, West Virginia, United States of America; 3 Department of Animal Science and Biotechnology, College of Agriculture and Life Science, Chungnam National University, Daejeon, Republic of Korea; University of Hawaii at Manoa, John A. Burns School of Medicine, United States of America

## Abstract

This study investigated the development of rat oocytes *in vitro* and *in vivo* following intracytoplasmic injection of heads from spermatozoa heat-dried at 50°C for 8 h and stored at 4°C in different gas phases. Sperm membrane and chromosome are damaged by the process of heat-drying. Oocyte activation and cleavage of oocytes were worse in oocytes injected with spermatozoa heat-dried and stored for 1 week than unheated, fresh spermatozoa, but in heat-dried spermatozoa, there were no differences in these abilities of oocytes between the samples stored in nitrogen gas and in air. The oocytes injected with heat-dried spermatozoa stored for 1 week could develop to the morula and blastocyst stages without difference between the samples stored in nitrogen gas and in air after artificial stimulation. Cleavage of oocytes and development of cleaved embryos were higher when heat-dried spermatozoa were stored for 3 and 6 months in nitrogen gas than in air. However, the ability of injected oocytes to develop to the morula and blastocyst stages was not inhibited even when heat-dried spermatozoa stored in both atmosphere conditions for as long as 6 months were used. When 2-cell embryos derived from oocytes injected with heads from spermatozoa heat-dried and stored for 1 week and 1 month were transferred, each 1 of 4 recipients was conceived, and the conceived recipients delivered 1 live young each. These results demonstrate that rat oocytes can be fertilized with heat-dried spermatozoa and that the fertilized oocytes can develop to term.

## Introduction

Very little information is available on intracytoplasmic sperm injection (ICSI) in rats, possibly because the large size of rat spermatozoa makes their microinjection into oocytes extremely difficult. In an early study, ICSI was performed using immature rat oocytes but the survival rate of injected oocytes was very low [1]. A similar low survival rate has also been reported using matured oocytes [2]. Such low survival of ICSI rat oocytes may be due to excessive amounts of medium that is injected into oocytes by using a relatively large bore pipette for injecting whole rat spermatozoa [3]. Thus, when microinjection of rat sperm heads was performed using piezo driven pipette with very small inner diameter, the survival rate of injected oocytes was increased and normal offspring derived from injected oocytes were obtained [4], [5].

ICSI into the ooplasm is an established technique in many mammalian species for producing live offspring when their spermatozoa lack motility, causing infertility. In fact, sperm immobilization significantly increases the rate of ICSI success [6]. This could perhaps be due to swift disintegration of sperm plasma membrane, and therefore swift intermingling of the sperm nucleus with the oocytes cytoplasm [7]. In cattle, isolated sperm heads have been used for ICSI with the successful production of viable blastocysts and normal live calves [8]. This report indicated that removal of the tail from the spermatozoa before injection did not affect development to the blastocyst stage in vitro. Furthermore, using such a technique, it has been reported that freeze-dried spermatozoa are able to produce normal blastocyst and live young when injected into oocytes in mouse [9]–[12], rabbit [13], bovine [14] and porcine [15]. These reports suggest that spermatozoa have lost their motility, acrosome integrity or plasma membrane integrity but have genetic integrity unmarred by drying. Yanagida et al. examined the thermostablity of mammalian sperm nuclei using ICSI method and reported that morphologically matured mammalian sperm nuclei do not lose the capability to form pronuclei or to synthesize DNA even when exposed to high temperature [16]. Somewhat, convective drying using an inert gas offer a simpler and less expensive alternative to freeze-drying without the requirement of any specialized equipment and the ability to perform the entire protocol at room temperature, and it is possible to store dried spermatozoa in a refrigerator or room temperature without using liquid nitrogen [17]. Recently, it has been reported that mouse oocytes with evaporative dried spermatozoa could develop to the blastocyst stage in vitro and live young after transplantation of two- to four-cell embryos [18]–[20]. Heat-dried sperm are similar to evaporative dried sperm on the technical approach.

In our previous study, it was demonstrated that bovine oocytes can be fertilized with heat-dried spermatozoa and that the fertilized oocytes can develop at least to the blastocyst stage [21]. However, in the study it was not examined whether the fertilized oocytes can develop to term *in vivo* after transplantation. The technique of embryo transfer is applicable much easier to small experimental animals than large domestic animals. This study was undertaken to evaluate the ability of heat-dried rat spermatozoa to support not only early embryonic development *in vitro* but also development to full term *in vivo* following injection into rat oocytes.

## Materials and Methods

### Media

Unless otherwise stated, all chemicals used in the present study were purchased from Sigma-Aldrich Chemical Co. (St. Louis, MO, USA). The medium used for heat-drying of cauda epididymal spermatozoa was 10 mM Tris-buffer (Nacalai Tesque, Kyoto, Japan) supplemented with 50 mM NaCl and 50 mM EGTA [ethylene glycol-bis(β-aminoethyl ether)-*N,N,N’,N’*-tetra acetic acid], referred to hereafter as EGTA Tris-HCl buffer solution. Modified Krebs Ringer bicarbonate (mKRB) solution, consisting of 94.6 mM NaCl, 4.78 mM KCl, 1.71 mM CaCl_2_, 1.19 mM KH_2_PO_4_, 1.19 mM MgSO_4_, 25.0 mM NaHCO_3_, 5.56 mM glucose (G-8270;), 21.58 mM sodium lactate (L-4263), 0.5 mM sodium pyruvate, 4 mg/ml fatty acid free BSA (A-6003), 75 µg/ml potassium penicillin G, 50 µg/ml streptomycin sulfate was used for preincubation of oocytes or early culture of oocytes after intracytoplasmic injection of sperm heads. The basic medium used for collection of oocytes and ICSI was mKRB containing lower concentration of NaHCO_3_ (5 mM) and 20 mM Hepes (mKRB-Hepes). The medium used for culture of embryos was modified rat one-cell embryo culture medium (mR1ECM) composed of 76.7 mM NaCl, 3.2 mM KCl, 2.0 mM CaCl_2_, 0.5 mM MgCl_2_, 25.0 mM NaHCO_3_, 10.0 mM sodium lactate, 0.5 mM sodium pyruvate, 7.5 mM glucose, 0.1 mM glutamine, 1 mg/ml polyvinylalcohol (PVA), 2% (v/v) minimal essential medium (MEM) essential amino acid solution (No. 11130-051; Gibco Laboratories, Grand Island, NY, USA), 1% (v/v) MEM nonessential amino acid solution (Gibco No. 11140-050).

### Heat-Drying of Spermatozoa

**Figure 1:**
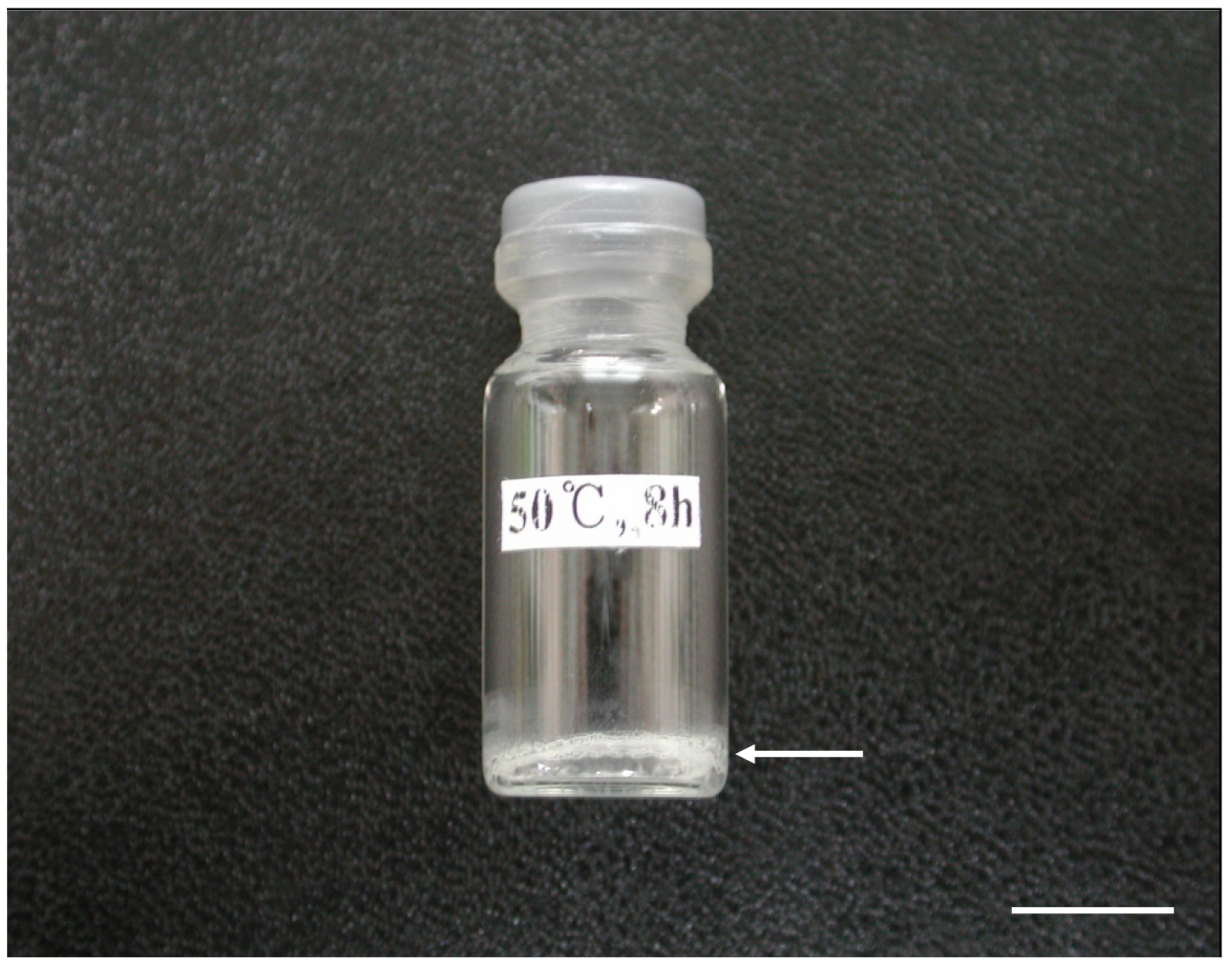
A bottle containing sperm sample heat-dried and stored for 1 week in nitrogen gas. A thin layer of the sperm sample adhering to the inner surface of the bottom of the bottle is visible (arrow). Bar = 1 cm.

Cauda epididymides were obtained from adult (4–8 months old) male Wister rats at room temperature. Spermatozoa were squeezed out from the epididymal ducts cut with a pair of fine scissor and a small drop of sperm mass was placed in EGTA Tris-HCl buffer solution (2–3 ml) in a 15-ml conical tube (Greiner Bio-One; Frickenhausen, Germany). After 5–10 min, spermatozoa were washed twice by centrifuging each at 250× g for 10 min. Sedimented spermatozoa were resuspended in 1 ml EGTA Tris-HCl buffer solution and 100 µl aliquots of the sperm suspension were transferred to 2 ml vial bottles (Maruemu Corporation, Ltd., Osaka, Japan). The bottles then were heated in a dry oven (MOV-212; Sanyo Electric Co., Ltd., Osaka, Japan) at 50, 56 and 90°C for various times. After heating, the bottles were closed quickly with rubber caps without removing air or exchanging air with nitrogen gas, firmly sealed with parafilm, and stored at 4°C for 7 days to 6 months (Fig. 1).

### Rehydration of Dried Spermatozoa

Heat-dried samples were rehydrated by adding 100 µl of sterile distilled water to the bottles and then transferred into 15 ml conical tubes. For washing spermatozoa, about 1 ml of mKRB-Hepes was added and centrifuged at 250× g for 10 min. Sedimented spermatozoa were resuspended in 1 ml mKRB-Hepes. The tubes were placed in ice water and the spermatozoa were sonicated for 10 sec (0.3 sec bursts at 0.7 sec interval/sec) using a 20% power output of a Branson Sonifier Model 250 (Branson Ultrasonics Co., Danbury, CT). The separation of heads from tails was successful in about 80% of spermatozoa. A portion (100 µl) of the sonicated suspension was transferred into a 1.5-ml tapered centrifuge tube containing 1 ml mKRB-Hepes and the diluted sperm suspension was centrifuged at 1,800× g for 3 min. The pelleted material, consisting of sperm heads and tails, was resuspended in 1 ml mKRB- Hepes.

### Evaluation of Acrosomal Membrane

To evaluate acrosomal membrane, the staining protocol was prepared as described previously [22] with minor modifications. Briefly, fresh and heat-dried sperm samples were smeared on microscope slide glass and dried for 30 min by air. These samples were then fixed with absolute ethanol for 30 min and treated with 100 µg/ml FITC-labelled peanut agglutinin (FITC-PNA) in PBS for 30 min on a 37°C warm plate. Following washing three times in PBS, these samples were stained with 1 mg/ml propidium iodide (PI) for 5 min and then evaluated using a scanning laser confocal microscope (LSM5 Live; Carl Zeiss, Germany) with Zeiss LSM5 Live Release ver. 4.2. SP1 Image Browser software. Acrosomal membrane and nucleus were stained green and red, respectively.

### Preparation of Oocytes

The protocol for current research was approved by the Research Ethics Committee of Okayama University. Immature 21- to 25-day-old female Wister rats were induced to superovulate by i.p. injections of 10–15 IU eCG (Serotropin: Teikoku-Zoki Co., Tokyo, Japan) and 10–15 IU hCG (Puberogen: Sankyo Co., Tokyo, Japan) 48 h later. The females were killed by cervical dislocation 15–16 h after hCG injection and the excised oviducts were placed in a small drop (100 µl) of mKRB-Hepes supplemented with 0.1% hyaluronidase. Oocytes with cumulus cells were released from the ampullar portion of the oviducts and kept in the medium for about 5 min. The oocytes freed from cumulus cells were washed in mKRB, placed into 100 µl of the same medium, and kept in a CO_2_ incubator (5% CO_2_ in air at 37°C) for 1 h until used for sperm injection.

### ICSI

The oocytes which had been kept in a CO_2_ incubator (37°C) were initially transferred into 100 µl mKRB-Hepes containing 5 µg/ml cytochalasin B to prevent spontaneous activation which occurs in rat oocytes at a high rate [23]. Then 5 µl medium containing 10–15 oocytes was placed in a petri dish cover (50×4 mm; Falcon No. 1006, Becton Dickinson Labware, Franklin Lakes, NJ, USA) and covered with paraffin oil (Nacalai Tesque Inc., Kyoto, Japan). A small drop (1 µl) of the suspension containing 20–40 sperm heads and some tails was introduced into a drop containing oocytes. Intracytoplasmic injection of sperm heads into oocytes was performed on a microwarm plate (MPF-10-N; Kitazato Supply Co., Ltd, Sizuoka, Japan) at 37°C at 200× magnification using a piezomicromanipulator controller (PMAS-CT150; Prime Tech, Tsukuba, Japan). The injection and holding pipettes were prepared from Borosilicate glass capillary tubes (Sutter Instrument Co. Novato, CA, USA) using a micropipette puller (P-97/IVF; Sutter Instrument Co., CA) and a microforge (MF-9; Narishige Co., Ltd, Tokyo, Japan). The external and internal diameters of the tip of the injection pipette were 4–5 µm and 2–3 µm, respectively. A sperm head was aspirated into the injection pipette so that the apex of sperm head was positioned facing the opening of the pipette. The tip of the pipette was brought in contact with the zona pellucida of the oocyte which was held by a holding pipette with an external diameter of 100–110 µm and an internal opening of 15–20 µm. The zona was drilled by applying two to three piezo pulses (intensity 2, speed 2). When the tip had reached into the ooplasm, a few piezo pulses were applied and a sperm head in a minimal amount of medium was expelled into the ooplasm; the pipette was then gently withdrawn from the oocyte. The injection procedure was completed within 50 min after introduction of sperm heads into a drop containing oocytes. A portion of sperm suspension without heating was used for ICSI as the control in one experiment. In that case, the final sonicated sperm suspension for ICSI was prepared with the same procedures as employed for obtaining dried samples. Sham injection was performed in a similar manner, with a minimum volume of medium expelled into the oocyte.

### Culture of ICSI Oocytes

In one experiment, the ICSI oocytes were washed three times with mKRB and cultured for 9–12 h in 100 µl of the same medium covered with paraffin oil in a culture dish (35×10 mm; Falcon No. 1008) at 37°C under 5% CO_2_ in air for examination of oocyte activation. For examination of cleavage and early development of ICSI oocytes, they were first cultured for 10 h in mKRB and then for various periods in mR1ECM.

In another experiments, the ICSI oocytes were washed three times with activation solution which consisted of 0.3 M mannitol, 0.1 mM CaCl_2_, 0.1 mM MgSO_4_ and 1 mg/ml fatty acid free BSA. Oocytes were then placed between two wire electrodes in the fusion chamber slide with 500 µl of activation solution. Using an electro cell manipulator (ECM 2001; BTX, Inc., San Diego, CA, USA), a direct current pulse of 1 kV/cm for 100 µsec was applied to oocytes, and then oocytes were washed three times with mKRB and cultured for 4 h in 100 µl of the same medium containing 2 mM 6-dimethylaminopurine (DMAP) covered with paraffin oil in a culture dish in a CO_2_ incubator (5% CO_2_ in air at 37°C). After the treatment, the oocytes were washed three times with mKRB, transferred into 100 µl of the same medium and cultured for 6 h and then for various periods in mR1ECM for examination of cleavage and early development of the treated ICSI oocytes.

### Examination of Activation and *In Vitro* Development of ICSI Oocytes

At 9–12 h after ICSI, oocytes were fixed with 2.5% (v/v) glutaraldehyde in phosphate beffer (pH 7.4) followed by 10% (v/v) neutral formalin and stained with 0.25% (w/v) lacmoid in 45% (v/v) acetic acid. The stained oocytes were examined for evidence of activation of oocytes and male pronucleus (MPN) formation using a phase-contrast microscope at a magnification of ×200 or ×400. The oocytes with an intact sperm head, an enlarged sperm head or a MPN were considered to have been successfully injected. Since it was difficult to distinguish the MPN from the female pronucleus, the oocytes with either two pronuclei and a second polar body or three pronuclei without a second polar body were considered to have a MPN. The oocytes with either one female pronucleus and a second polar body or two female pronuclei without a second polar body were considered to be activated. For evaluation of developmental potential, oocytes having two pronuclei 10 h after injection were washed three times with mR1ECM, cultured in the same medium (100 µl) at 37°C under 5% CO_2_ in air, and examined for their cleavage, development to the 4-cell, morula and blastocyst stages 24, 72, 96 and 120 h after ICSI, respectively, under a dissecting microscope.

### Evaluation of Chromosomal Complements

Karyotype analysis was carried out to investigate chromosomal complements. Briefly, zygotes were incubated with 50 ng/ml colcemid (Invitrogen) at 37°C for 4 h and transferred to hypotonic solution (75 mM KCl) for 20 min. Zygotes were then fixed in the fixative solution (methanol/glacial acetic acid 3∶1) and dropped onto a slide glass. The fixed zygotes were kept at 4°C until ready for spreads. The slide glass was air-dried, then aged at 90°C for 30 min, stained with Giemsa, and observed using a stereoscopic microscope (E600, Nikon, Japan).

### Assessment of developmental potential of ICSI Oocytes

Injected oocytes that had reached to the 2-cell stage 24 h after injection were transferred into recipient female Wistar rats (2–4 months old). Pseudopregnancy was induced in the female rats by inserting a glass rod, connected to an electric vibrator, into their vaginae between 18∶00 and 19∶30 h on the day of proestrus as assessed from the vaginal smear (Day 0). Between 14∶00 and 16∶00 h on the following day (Day 1) the females were anesthetized with an i.p. injection of Avertin (0.012 ml/g body weight; Aldrich Chemical Co., Inc., Milwaukee, WI, USA), and the ovary and oviduct were exposed through a dorsal incision. To prevent bleeding, a small drop of 0.1% epinephrine solution was put on the surface of the bursal membrane, and then a small cut was made on the bursal membrane using two pairs of watchmaker’s forceps. The embryos were picked up with a mouth-controlled pipette with curved tip (150–200 µm in diameter). The tip of the pipette was inserted into the fimbriated ostium and the contents were delivered by gentle pressure. Eight to seventeen embryos were transferred into each oviduct. After transfer, the vaginal smear of the recipients was examined daily. Recipients that showed proestrous or estrous were killed and their uterine horns were examined for implantation sites. Pregnant females were allowed to give birth and were then killed to examine their uterine horns for implantation sites. The young obtained from embryos derived from oocytes injected with heat-dried sperm heads were nursed by foster mothers.

### Statistical Analysis

All proportional data on activation and cleavage of ICSI oocytes and development of embryos obtained from 4–6 replicated experiments were subjected to an arc-sine transformation, and the transformed values were analyzed using one-way ANOVA. When ANOVA revealed a significant effect, the treatments were compared by Fisher’s protected least significant difference (LSD) test.

## Results

### Appearance of Rehydrated Spermatozoa after Heat Drying

**Figure 2:**
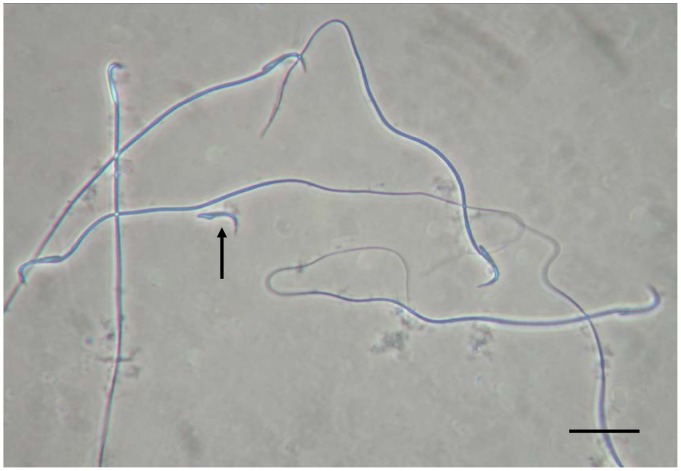
A Phase-contrast micrograph of spermatozoa dried and rehydrated after storage at 4°C for 1 week in nitrogen gas. Many heat-dried spermatozoa have wavy tails. A sperm head (arrow) separated from a tail is also visible. Bar = 20 µm.

**Figure 3:**
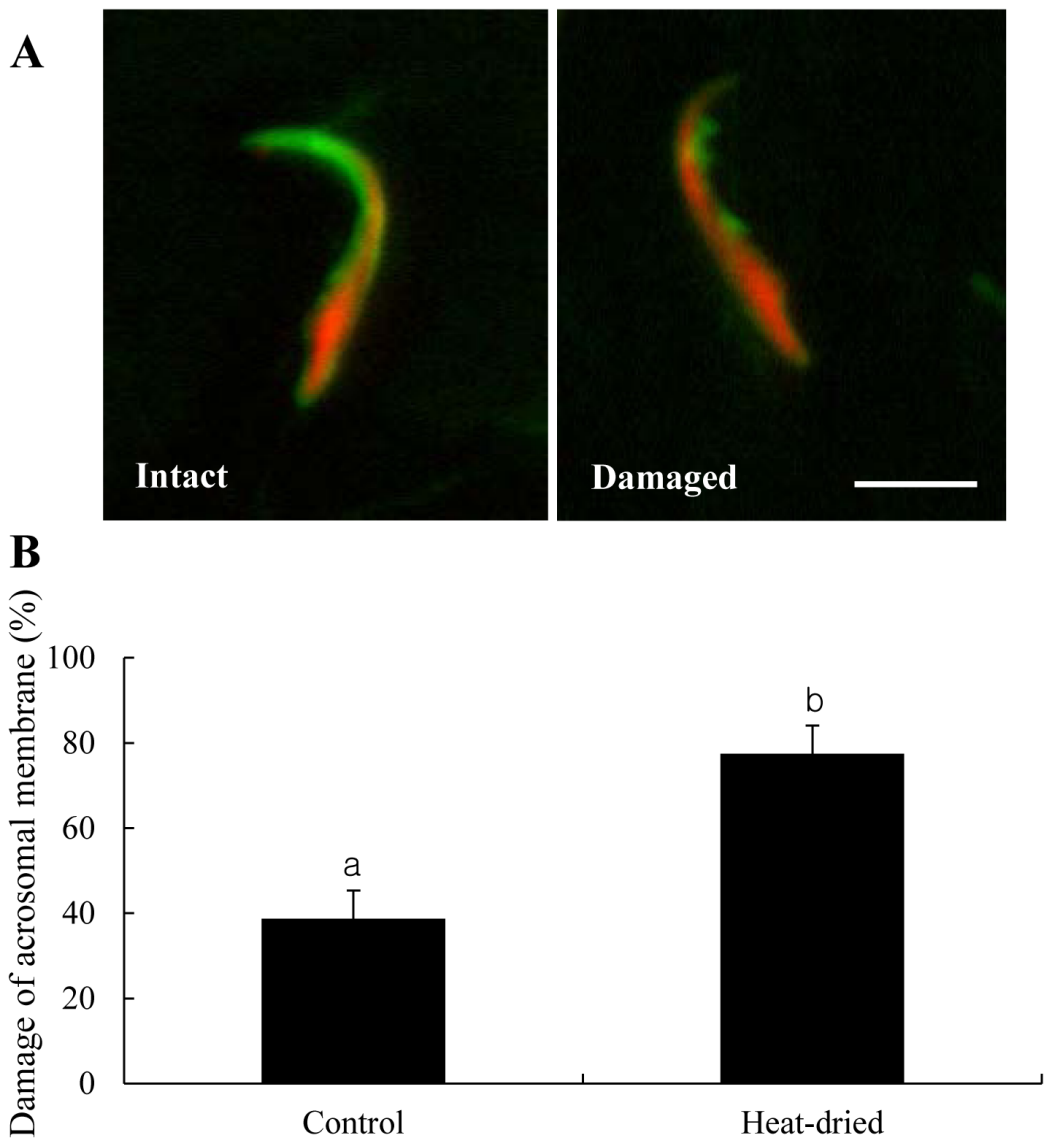
Acrosomal membrane damage of the rat sperm head. (A) Spermatozoa were sonicated to separate head and tail. Acrosome and nucleus of sperm head were stained with green (FITC-PNA) and red (PI), respectively. Acrosomal membrane remains “intact” or “damaged”. Bar = 5 µm. (B) Sperm heads that were not heat-dried (control) or heat-dried were randomly counted. Experiments were repeated separately four times using 20 sperm heads per replicate. The values are expressed as mean ± SEM. Values with different letters within each category differ significantly (P<0.05).

When heat-dried, about 60% of rehydrated spermatozoa had wavy tails, and about in 20% of sperm heads were separated from tails (Fig. 2): these proportions were not different either between different storage conditions (in air and nitrogen gas) or among different storage periods (1 week, and 1, 3 and 6 months). Acrosomal membrane staining was performed to examine whether the sperm membrane is damaged by the process of heat-drying (Fig. 3A). The proportion of acrosomal membrane damage was significantly increased in heat-dried spermatozoa than in control (Fig. 3B).

### *In Vitro* Development of Oocytes Injected with Heads from Spermatozoa Heat-dried at Various Temperature and Stored at 4°C for 1 week in Air

**Figure 4:**
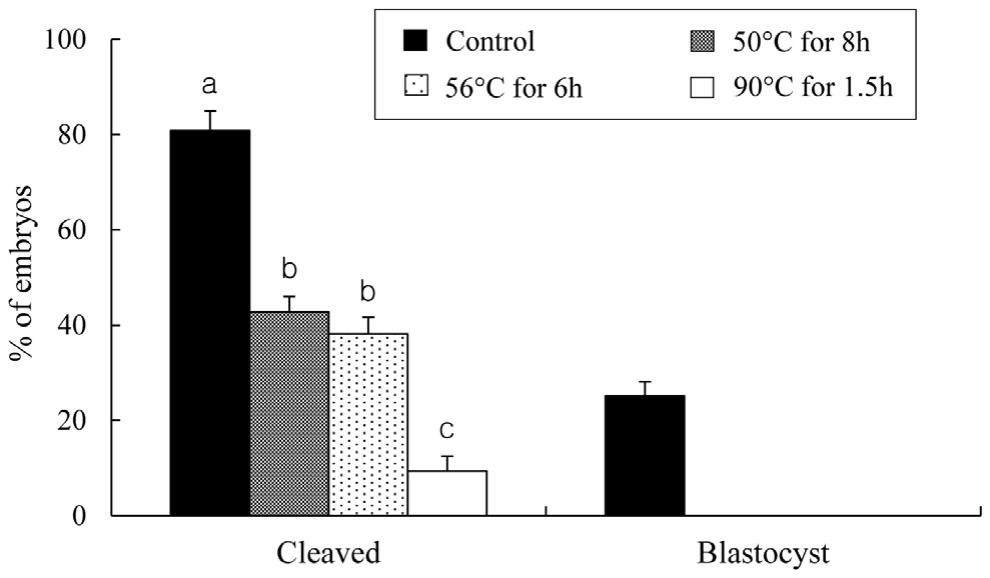
In vitro development of rat oocytes injected with heads from spermatozoa that were not heat-dried (control) or heat-dried at various temperature with various times and stored at 4°C for 1 week in air. Experiments in each treatment were repeated separately four times using 20–25 oocytes per replicate. Injected oocytes were cultured and examined 24 and 120 h after the start of culture for 2-cell stage and blastocysts, respectively. The values are expressed as mean ± SEM; the total number of oocytes cultured after injection was 99, 98, 94, 84 and 83 for examination of development for spermatozoa unheated (control), dried at 50, 56, 90 and 120°C, respectively. Values with different letters within each category differ significantly (P<0.05).

As shown in Fig. 4, the proportion of cleavage was significantly increased when spermatozoa dried at 50 and 56°C, compared with 90, were used. Especially, oocytes injected with heads from spermatozoa heat-dried at 50°C for 8 h were showed the highest proportion of cleavage even though 50 and 56°C has no significant difference. No blastocyst was observed in the groups injected with heat-dried sperm heads. Based on the result obtained in Fig. 4, spermatozoa heat-dried at 50°C for 8 h was introduced for the next experiments.

### Chromosomal Damage of Heat-dried Spermatozoa

**Figure 5:**
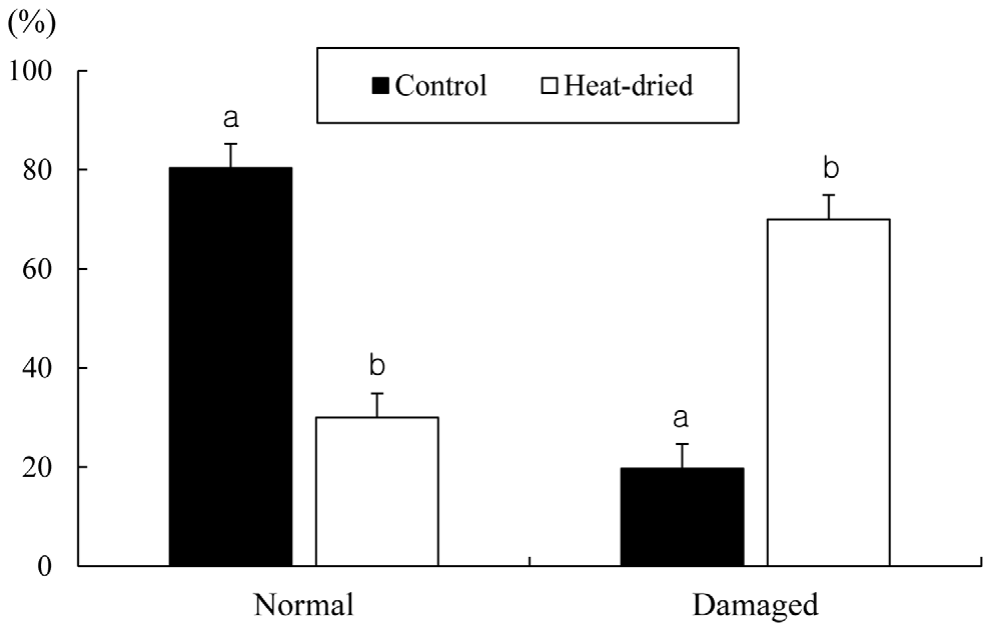
Chromosomal analysis of rat oocytes injected with heads from spermatozoa that were not heat-dried (control) or heat-dried at 50°C for 8 h and stored at 4°C for 1 week in air. Experiments in each treatment were repeated separately four times using 10–13 oocytes per replicate. Chromosomes were evaluated as “normal” or “damaged”. The values are expressed as mean ± SEM. Values with different letters within each category differ significantly (P<0.05).

As shown in Fig. 5, when oocytes injected with heads from spermatozoa heat-dried at 50°C for 8 h and stored at 4°C for 1 week in air, the proportion of chromosomal damage was significantly increased in heat-dried spermatozoa than in control (70.0% vs. 19.7%). This result indicated that rat chromosomes are damaged by the process of heat-drying.

### *In Vitro* Development of Oocytes Injected with Heads from Spermatozoa Heat-dried and Stored at 4°C for 1 week in Nitrogen gas or in Air

**Figure 6:**
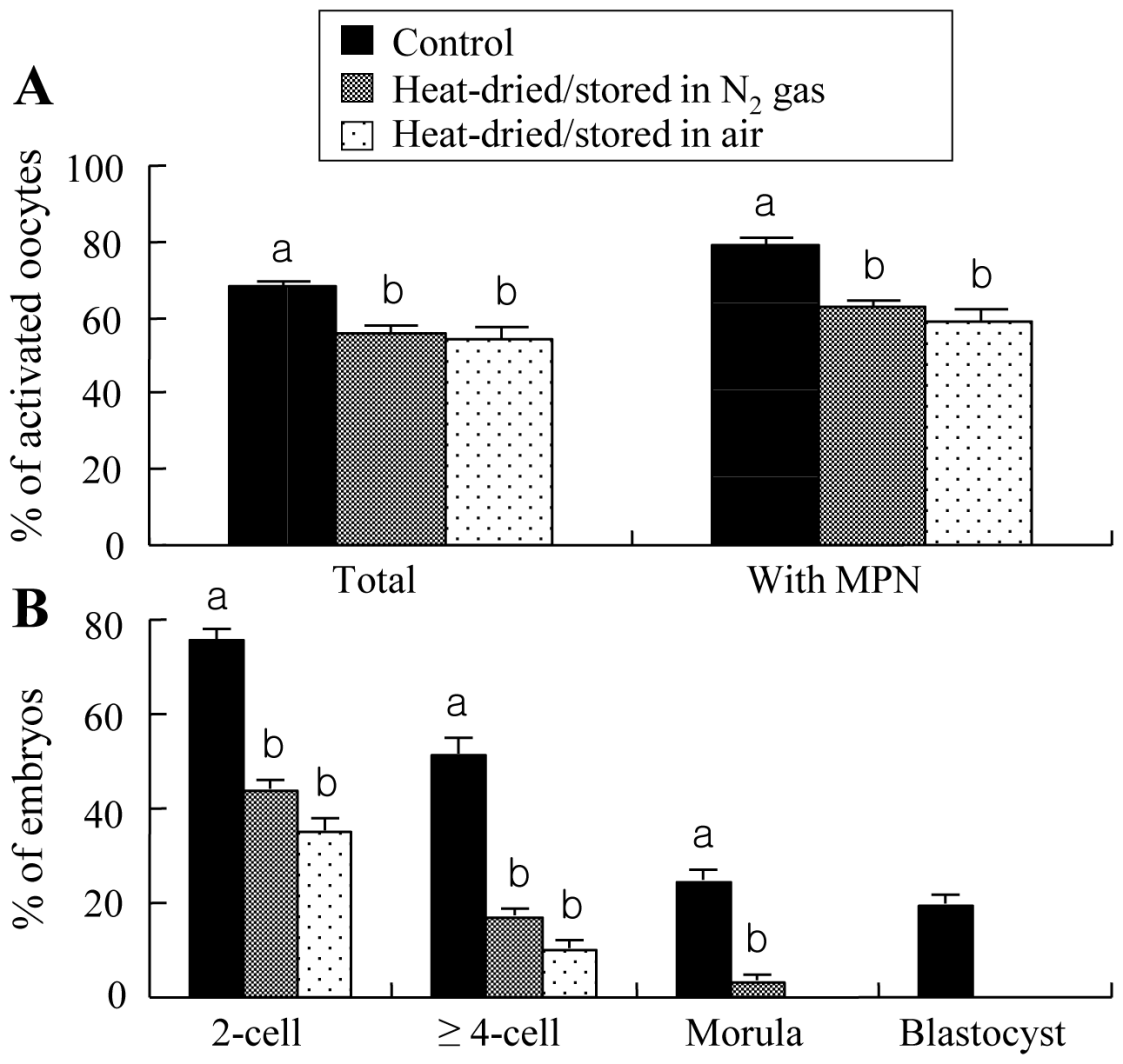
Activation (A) and in vitro development (B) of rat oocytes injected with heads from spermatozoa that were not heat-dried (control) or heat-dried at 50°C for 8 h and stored at 4°C for 1 week in nitrogen gas or in air. Experiments in each treatment were repeated separately six times using 15–20 oocytes per replicate. Injected oocytes were examined 9–12 h after injection for activation, and those with two pronuclei obtained 10 h after injection were cultured and examined 24, 72, 96 and 120 h after the start of culture for 2-cell stage, ≥4-cell stage, morulae and blastocysts, respectively. The values are expressed as mean ± SEM; the total number of oocytes cultured after injection was 107, 103 and 97 for examination of activation and 98, 101 and 96 for examination of development for spermatozoa unheated (control), stored in nitrogen gas and in air after heat drying, respectively. Values with different letters within each category differ significantly (P<0.05). MPN: male pronucleus.

As shown in Fig. 6A, injection of dried spermatozoa resulted in significantly less activated oocytes and MPN formation in activated oocytes than obtained following injection of unheated, fresh spermatozoa. However, no significant differences in these values between dried spermatozoa stored in air and nitrogen gas. Significantly less oocytes also cleaved to the 2-cell and ≥4-cell stages when spermatozoa heat-dried, compared with unheated, were used, but no significant differences in cleavage rates between dried spermatozoa stored in air and nitrogen gas (Fig. 6B). When stored in nitrogen gas, a small proportion of oocytes injected with dried spermatozoa developed to the morula stage, but no development to the stage was observed when dried spermatozoa were stored in air. No blastocyst formation was observed when spermatozoa were dried, irrespective of the storage conditions.

### *In Vitro* Development of Oocytes Induced Activation after being Injected with Heads from Spermatozoa Heat-dried and Stored at 4°C for 1 week in Nitrogen Gas or in Air

**Figure 7:**
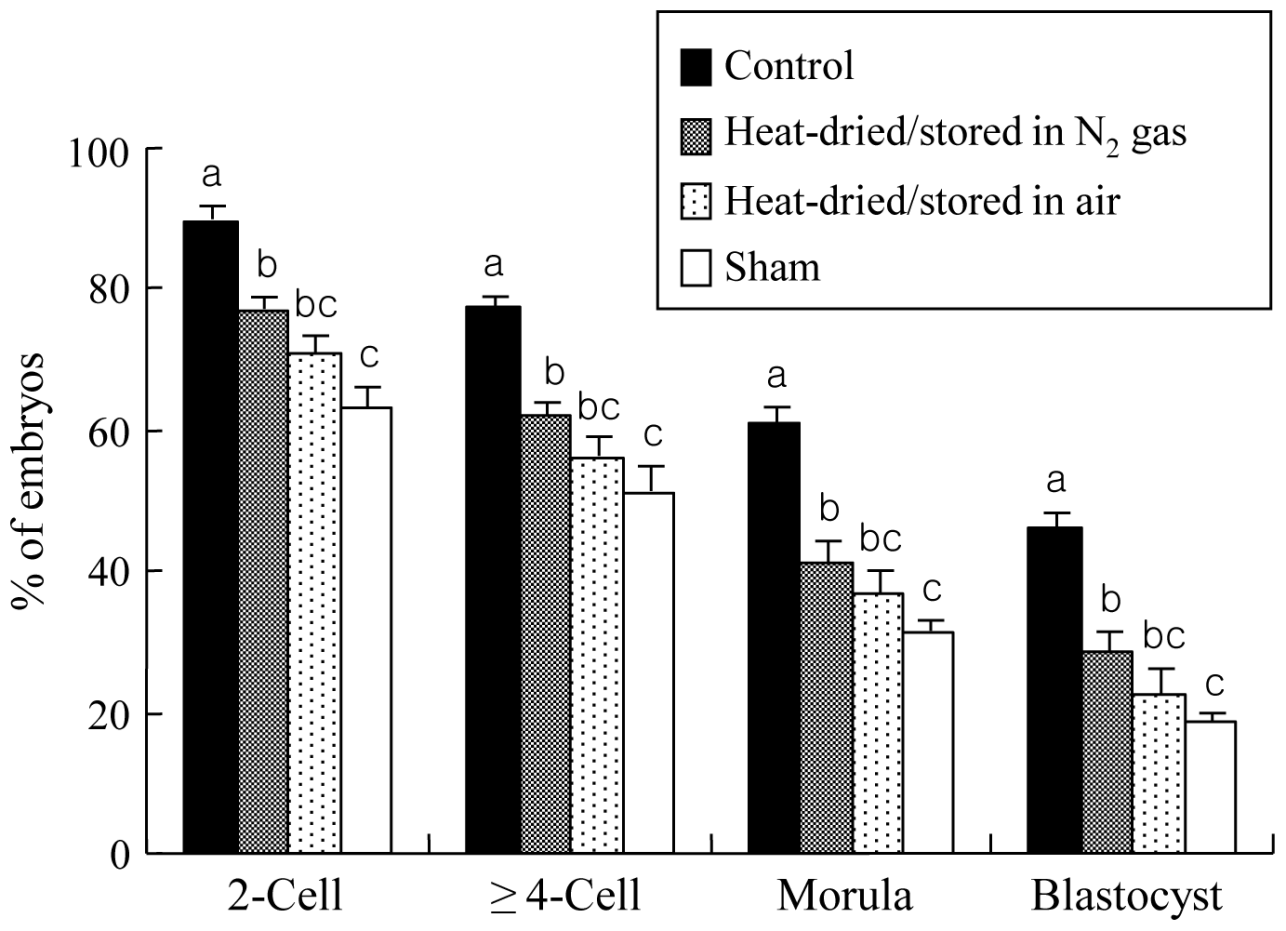
In vitro development of rat oocytes stimulated with a single direct current pulse (1 kV/cm) for 100 µsec+DMAP after being injected with heads from spermatozoa that were not heated (control) or heat-dried at 50°C for 8 h and stored at 4°C for 1 week in nitrogen gas or in air. Experiments in each treatment were repeated separately six times using 15–20 oocytes per replicate, along with sham injection of six to seven oocytes. Oocytes with two pronuclei (sperm injection) or one or two pronuclei (sham injection) obtained 10 h after injection were cultured and examined 24, 72, 96 and 120 h after the start of culture for 2-cell stage, ≥4-cell stage, morulae and blastocysts, respectively. The values are expressed as mean ± SEM; the total number of oocytes cultured after injection was 97, 102 and 93 for examination of development for spermatozoa unheated (control), stored in nitrogen gas and in air after heat drying, respectively. Values with different letters within each category indicate significant difference (P<0.05).

As shown in Fig. 7, oocytes injected with heat-dried spermatozoa could develop to the blastocyst stage when they were treated with a single direct current pulse+DMAP after injection. However, significantly fewer oocytes cleaved to the 2-cell and ≥4-cell stages and developed to the morula and blastocyst stages when spermatozoa heat-dried, compared with unheated, were used. Although there were no significant differences in these values between heat-dried spermatozoa stored in nitrogen gas and in air, the values in the sample stored in nitrogen gas, but not in air, were significantly higher compared with those obtained in sham injection.

### *In Vitro* Development of Oocytes Induced Activation after being Injected with Heads from Spermatozoa Heat-dried and Stored at 4°C for Various Periods in Nitrogen Gas or in Air

**Figure 8:**
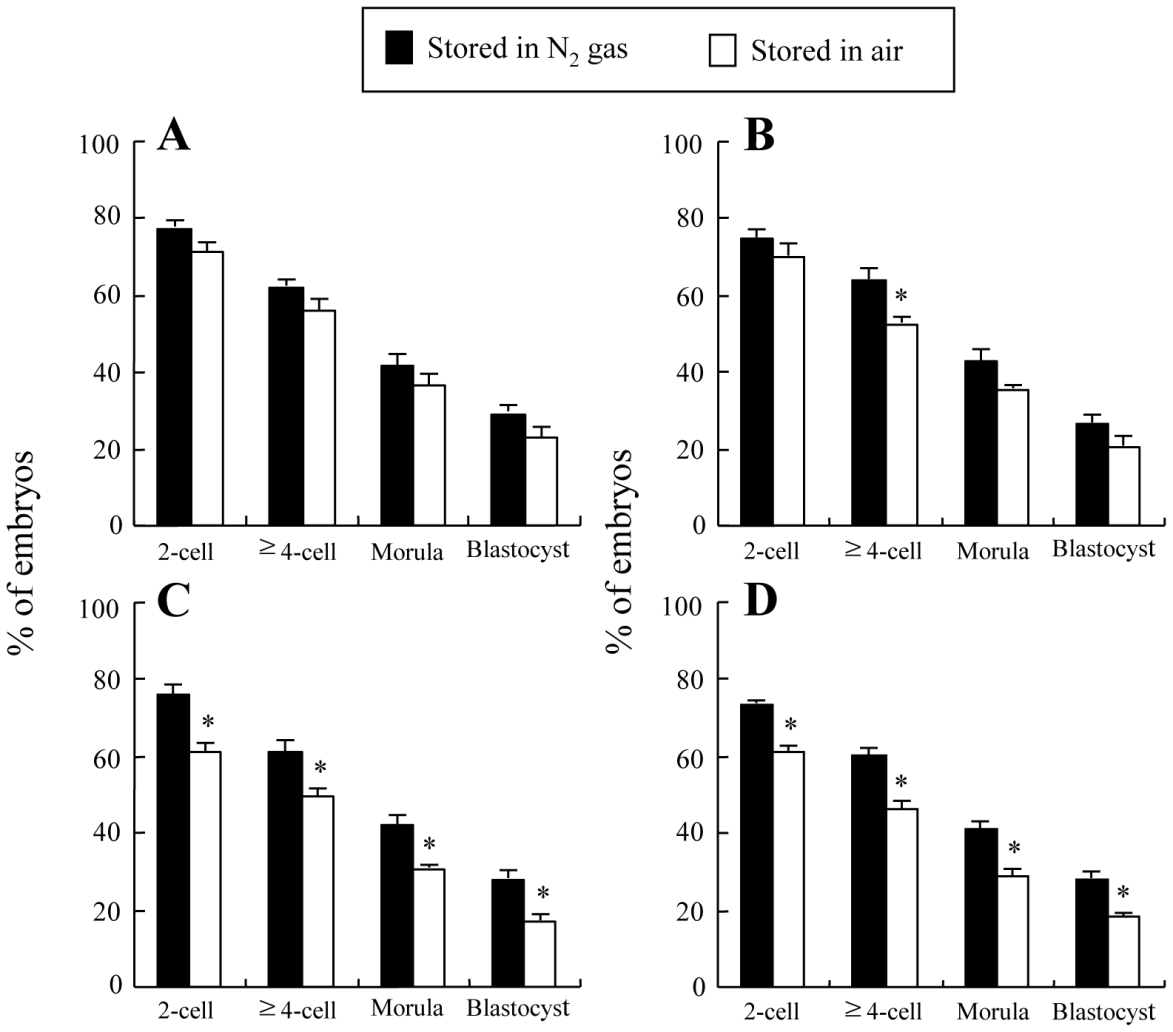
In vitro development of rat oocytes stimulated with a single direct current pulse (1 kV/cm) for 100 µsec+DMAP after being injected with heads from spermatozoa heat-dried at 50°C for 8 h and stored at 4°C for 1 week (A), and 1 (B), 3 (C) and 6 months (D) in nitrogen gas or in air. Injected oocytes with two pronuclei obtained 10-cell stage, ≥4-cell stage, morulae and blastocysts, respectively. Experiments in each treatment were repeated separately six and five times for spermatozoa stored for 1 week and 1–6 months, respectively, using 15–20 oocytes per replicate. The values are expressed as mean ± SEM; the total number of oocytes cultured after injection was 102, 83, 83 and 78 for spermatozoa stored in nitrogen gas and 93, 88, 89 and 92 for spermatozoa stored in air, each for 1 week, and 1, 3 and 6 months of storage, respectively. *P<0.05 compared with N_2_ gas stored group.

When injected with heat-dried spermatozoa that were stored for 1 week and 1 month, there were generally no significant differences in developmental competence of injected oocytes up to the blastocyst stage between the samples stored in nitrogen gas and in air (Fig. 8A, B). Only an exception was observed in cleavage to the ≥4-cell stage in the sample stored for 1 month (Fig. 8B), in that case cleavage rate was significantly higher in the sample stored in nitrogen gas than in air. When heat-dried spermatozoa were stored for 3 and 6 months, the proportions of injected oocytes cleaved to the 2-cell and ≥4-cell stages and developed to the morula and blastocyst stages were all significantly higher in the sample stored in nitrogen gas than in air (Fig. 8C, D). About 28% of injected oocytes could develop to the blastocyst stage even when heat-dried spermatozoa stored as long as for 6 months in nitrogen gas were used (Fig. 8D).

### *In Vivo* Development of Oocytes Injected with Heads from Spermatozoa Heat-dried and Stored at 4°C for Various Periods in Nitrogen Gas

**Figure 9:**
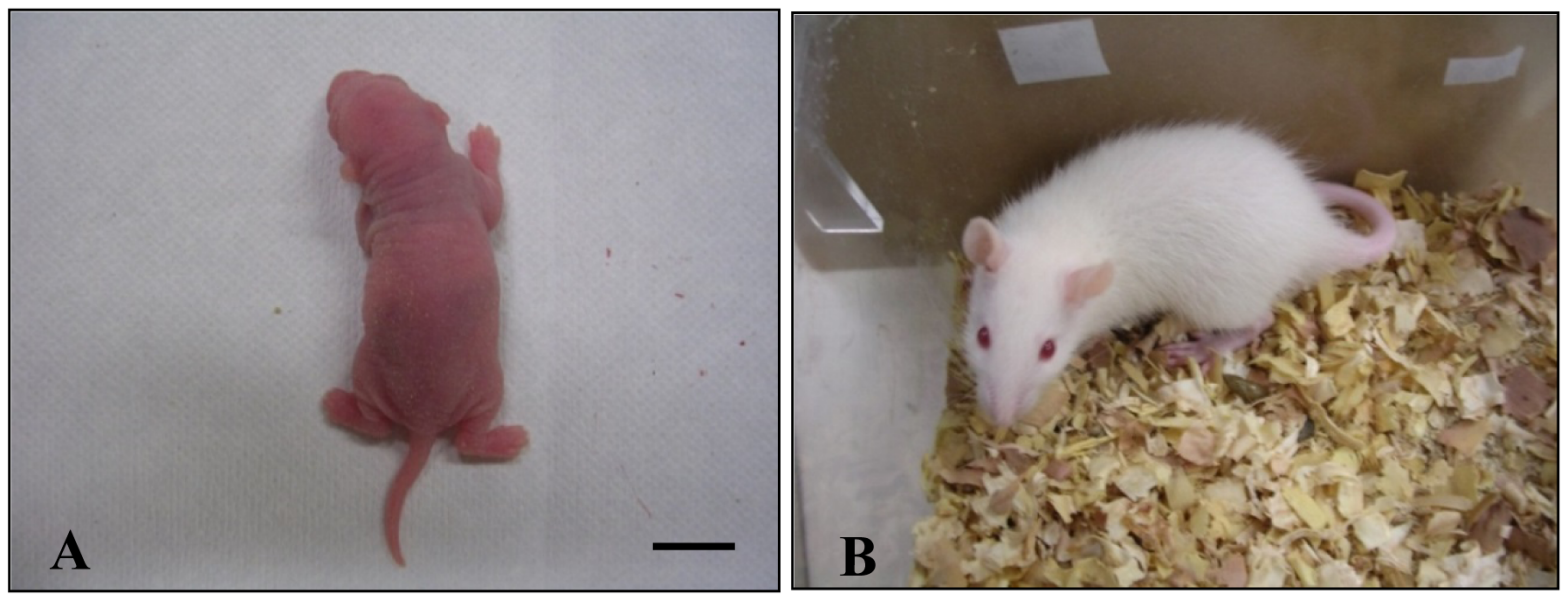
A live young from rat heat-dried spermatozoa. (A) A rat live-born young, at 1 day old, obtained after transplantation of 2-cell embryos that were produced from oocytes injected with heat-dried spermatozoa stored at 4°C for 1 week in nitrogen gas. (B) The young at 30 days old. Bar = 1 cm.

When 2-cell stage embryos derived from oocytes injected with heads from spermatozoa heat-dried and stored for 1 week and 1 month were transferred, each 1 of 4 recipients was conceived, and the conceived recipients delivered 1 live young each (Table 1). The young (male) derived from spermatozoa stored for 1 week (Fig. 9A) grew normally in appearance at least to 30 days old (Fig. 9B). However, the young (female) derived from spermatozoa stored for 1 month died on the next day of delivery. No recipients were conceived when heat-dried spermatozoa stored at 3 and 6 months were used for injection. Implantation rates were significantly lower when heat-dried than unheated, fresh spermatozoa were used for injection. In heat-dried spermatozoa, significantly fewer embryos implanted when the samples stored for 3 and 6 months than 1 week and 1 month were used.

**Table 1:** In vivo development of rat 2-cell embryos produced from oocytes that were injected with heads from spermatozoa heat-dried and stored for various periods in nitrogen gas and induced activation by a single direct current pulse (1 kV/cm) for 100 µsec+DMAP.

Spermatozoa	Storageperiods	No. of recipients(conceived/transferred)		No. of embryostransferred	No. (%) ofimplantation sites	No. (%) ofyoung born
Control^1^	–	2/5		113	37 (33.0)^a^	7 (6.6)^2^
Heat-dried	1 week		1/4	97	22 (22.5)^b^	1 (1.0)
	1 mo	1/4		100	23 (23.2)^b^	1 (1.0)^3^
	3 mo	0/4		102	12 (11.9)^c^	0
	6 mo	0/3		99	11 (11.2)^c^	0

a–c Values with different superscripts within column differ significantly (P<0.05).

1 Control (unheated) was not activated by a single direct current pulse (1 kV/cm) for 100 µsec+DMAP.

2 One young died 7 days after delivery.

3 The young died on the next day of delivery.

## Discussion

The present study has demonstrated for the first time that rat oocytes can be activated following intracytoplasmic injection of sperm heads isolated from heat-dried spermatozoa, and male pronuclei can be formed in the activated oocytes. Although injected oocytes were generally difficult to develop to the blastocyst stage when injected oocytes were not induced artificial activation, about 23–29% of oocytes developed to the blastocyst stage when they were induced artificial activation after being injected with heat-dried sperm heads that were stored at 4°C for 1 week in nitrogen gas or in air. The development to the blastocyst stage observed especially in oocytes injected with spermatozoa stored in nitrogen gas after heat-drying does not appear to be parthenogenetic, because the value (about 29%) obtained in the sample stored in nitrogen gas, but not in air, was higher compared with that obtained in sham injection. Furthermore, the development to term of oocytes injected with spermatozoa heat-dried and stored in nitrogen gas demonstrates that blastocysts from the sperm-injected oocytes were not parthenotes.

Neither the injection process nor the spermatozoon/sperm head are sufficient to activate rat oocytes following ICSI. The developmental rate of rat oocytes injected even with fresh spermatozoa is low [4], [5]. This is in contrast to mice in which oocytes injected with evaporative dried spermatozoa are readily activated without artificial stimulation [17]–[20]. It is suggested that chromosomal damage and deterioration of the sperm-borne oocyte-activating factor (SOAF) [24] may be induced during the heat-drying process of rat spermatozoa. In the present study, rat oocytes injected with heat-dried spermatozoa developed to the blastocyst stage when they were treated with a single direct current pulse+DMAP after being injected. This indicates that deterioration of SOAF may be minimized by such treatment. Furthermore, more than 50% of rat oocytes developed parthenogenetically to the blastocyst stage when they were treated with repeated electric current pulses and then with DMAP [25]. Interestingly, rat chromosomes are damaged by the process of heat-drying in the present study. Hochi et al. [26] reported 65% chromosomal damage when freeze-dried spermatozoa stored at 4°C for one year. Chromosomal damage originated from heat-dried spermatozoa was higher than freeze-dried spermatozoa. This result support that embryonic developmental competence of oocytes injected with heat-dried spermatozoa was lower than freeze-dried spermatozoa [14], [21].

In the present study, cleavage to the 2- to 4-cell stages and development to the morula and blastocyst stages were higher in injected oocytes stored for long period (3 and 6 months) in nitrogen gas than in air. This may be due to that spermatozoa were protected from being oxidized. Although it is unknown whether chromosomal integrity and SOAF activity are affected by oxidation, exposure of cells with damaged membranes to radical oxygen species can result in loss of DNA integrity [27].

However, development of rat oocytes produced with heat-dried spermatozoa was significantly less compared with those produced with fresh spermatozoa even after injected oocytes were induced artificial activation. During normal fertilization, the sperm membrane does not enter the ooplasm. Removal of sperm plasma membrane accelerates the activation of injected mouse oocytes [28]. Moreover, membrane-damaged spermatozoa improves sperm head decondensation and production of blastocysts in ICSI oocytes in humans [29] and pigs [30]. Removal of the sperm plasma membrane may facilitate mixing of nuclear and ooplasmic material [31]. It was examined whether the sperm acrosomal membrane is damaged by the process of heat-drying in the present study, it is possible that sperm plasma and other membranes may be injured or removed by the additional treatment, the sonication, of rehydrated spermatozoa to separate into heads and tails. Thus, the injection of sperm heads isolated from heat-dried spermatozoa may accelerate the development of activated oocytes. Nevertheless, the incidence of early development of oocytes was significantly less when injected with heat-dried than fresh sperm heads that may have less injured sperm membranes. In rabbits, the process of freeze-drying may induce chemical changes in the sperm plasma membranes [32]. The process of heat-drying may induce the same changes in the plasma membrane by which dissolution of the membranes becomes more difficult compared with fresh spermatozoa. On the other hand, during normal fertilization, the vesicle-associated membrane proteins (VAMP) are lost from the acrosome-reacted spermatozoa, and the nuclear matrix proteins (NuMA) appear in the decondensing sperm heads [33]. In the rhesus monkey, injection of acrosome-intact sperm heads resulted in a delay of sperm head decondensation [34]. These authors suggested that persistence of VAMP in sperm heads is responsible for the delay of nuclear decondensation. Persistence of VAMP in heat-dried sperm heads may not be responsible for lower developmental ability of rat oocytes injected with heat-dried than fresh sperm heads, because fresh sperm heads may have intact acrosome.

In the present study, it was demonstrated that rat oocytes injected with heads from heat-dried spermatozoa develop to term after transfer into recipients. Hoshi et al. [35] have reported that rabbit oocytes injected with spermatozoa heat-treated at 60°C for 30 min cleaved and developed to the 6- to 8-cell stages. Production of live offspring derived from mouse oocytes injected with spermatozoa heat-treated at 56°C for 30 min has also been reported [36]. Thus, mammalian spermatozoa can retain their genetic integrity not only after heating but also after drying by heating. Furthermore, since a sperm centrosome is absent in sperm heads isolated from tails by sonication, the results of the present study indicate that the sperm centrosome is not necessary for normal development to term of rat oocytes as reported by Said et al. [5]. This has also been reported in cattle [37], pigs [38], mice [39] and hamsters [40].

In conclusion, the present study demonstrated that rat spermatozoa, dried by heating and used for ICSI, induce oocyte activation and subsequent embryonic development in vitro. It was also demonstrated that injected oocytes develop to term following embryo transfer. Therefore, heat drying might provide a very simple method of preserving rat spermatozoa for reasonable lengths of time.
